# Innovative use of data sources: a cross-sectional study of data linkage and artificial intelligence practices across European countries

**DOI:** 10.1186/s13690-020-00436-9

**Published:** 2020-06-10

**Authors:** Romana Haneef, Marie Delnord, Michel Vernay, Emmanuelle Bauchet, Rita Gaidelyte, Herman Van Oyen, Zeynep Or, Beatriz Pérez-Gómez, Luigi Palmieri, Peter Achterberg, Mariken Tijhuis, Metka Zaletel, Stefan Mathis-Edenhofer, Ondřej Májek, Håkon Haaheim, Hanna Tolonen, Anne Gallay

**Affiliations:** 1 Department of Non-Communicable Diseases and Injuries, Santé Publique France, 12 rue du Val d’Osne, 94415 Saint-Maurice, France; 2 Epidemiology and public health, Sciensano, Brussels, Belgium; 3 Health information centre, Institute of hygiene, Vilnius, Lithuania; 4 Department of public health, Ghent University, Ghent, Belgium; 5 Institute of research and information for health economics, Paris, France; 6 National Centre for Epidemiology & CIBERESP, Carlos III Institute of Health, Madrid, Spain; 7 Department of Cardiovascular, Endocrine-metabolic Diseases and Aging, National Institute of Health, Rome, Italy; 8 National Institute for Public Health and the Environment (RIVM), Bilthoven, The Netherlands; 9 National Institute of Public Health (NIJZ), Ljubljana, Slovenia; 10 The Austrian National Public Health Institute (Gesundheit Österreich GmbH, GÖG), Vienna, Austria; 11 Institute of Health Information and Statistics of the Czech Republic, Prague, Czech Republic; 12 Institute of Biostatistics and Analyses, Faculty of Medicine, Masaryk University, Brno, Czech Republic; 13 The Norwegian Directorate of Health, Oslo, Norway; 14 Finnish Institute for Health and Welfare (THL), Helsinki, Finland

**Keywords:** Innovation, Linked data, Artificial intelligence, Machine learning technique, Health status monitoring, Public health surveillance, Health information, Health indicators

## Abstract

**Background:**

The availability of data generated from different sources is increasing with the possibility to link these data sources with each other. However, linked administrative data can be complex to use and may require advanced expertise and skills in statistical analysis. The main objectives of this study were to describe the current use of data linkage at the individual level and artificial intelligence (AI) in routine public health activities, to identify the related estimated health indicators (i.e., outcome and intervention indicators) and health determinants of non-communicable diseases and the obstacles to linking different data sources.

**Method:**

We performed a survey across European countries to explore the current practices applied by national institutes of public health, health information and statistics for innovative use of data sources (i.e., the use of data linkage and/or AI).

**Results:**

The use of data linkage and AI at national institutes of public health, health information and statistics in Europe varies. The majority of European countries use data linkage in routine by applying a deterministic method or a combination of two types of linkages (i.e., deterministic & probabilistic) for public health surveillance and research purposes. The use of AI to estimate health indicators is not frequent at national institutes of public health, health information and statistics. Using linked data, 46 health outcome indicators, 34 health determinants and 23 health intervention indicators were estimated in routine. The complex data regulation laws, lack of human resources, skills and problems with data governance, were reported by European countries as obstacles to routine data linkage for public health surveillance and research.

**Conclusions:**

Our results highlight that the majority of European countries have integrated data linkage in their routine public health activities but only a few use AI. A sustainable national health information system and a robust data governance framework allowing to link different data sources are essential to support evidence-informed health policy development. Building analytical capacity and raising awareness of the added value of data linkage in national institutes is necessary for improving the use of linked data in order to improve the quality of public health surveillance and monitoring activities.

## Background

The availability of administrative data generated from different sources is increasing. The possibility to link these data sources with other databases offers unique opportunities to answer those research questions, which require a large sample size or detailed data on hard-to-reach populations. This methodology link available information from different sources and can generate evidence at population level with a high level of external validity and relevance for policy making [1]. Over an extensive period, data linkage ensures a high statistical power, thereby reducing methodological issues relating to attrition, recall bias and lost-to-follow up [2]. This technique also allows performing more detailed stratified analyses of subgroups according to age, or specific geographical regions, and providing rapid access to data collected in a standardized format [3–5].

The value of any surveillance system ultimately depends on timely and reliable information [6]. There are several data sources, which are used for public health surveillance, for example, health interviews and examination surveys, diseases-specific registries, epidemiological cohort studies, hospital discharge data, health insurance claims, mortality database, etc. Traditional data sources (e.g., health interview and examination surveys, disease-specific registries, etc.) and administrative data sources (e.g., hospital discharge, health insurance claims, causes of mortality data, etc.) complement each other and can increase the completeness and comprehensiveness of health information by taking into account various dimensions of health and risk factors influencing health status directly and indirectly.

Linking various data sources improves the completeness and comprehensiveness of information to guide health policy process, effective patient care and health services management [7]. Data linkage is an important technique that connects detailed information about individuals or entities from different data sources to enrich or create new data source. This methodology potentiate the capacity to study disease burden and progression, risk factors, care pathways and long-term outcomes for public health research and health surveillance [1]. Many countries have already invested in data linkage to improve their health information system [8], but there are wide differences in capacity across European countries to perform data linkage in routine. However, linked administrative data can be complex to use and may require advanced expertise and skills in statistical analysis [9]. Generating efficiently comparable and timely health information across European Union (EU), European Economic Area (EEA) and other European countries requires skills and expertise in performing data linkage and applying AI to estimate health indicators. Artificial intelligence (AI), also known as machine intelligence, is a branch of computer science that aims to imbue software with the ability to analyze its environment using either predetermined rules and search algorithms, or pattern recognizing machine learning models, and then make decisions based on those analyses [10]. Machine learning is an application of AI and is often applied for the diagnosis of certain medical conditions as well as outcome prediction and prognosis evaluation with high precision [11].

We explored the differential use of data linkage in routine health monitoring based on the latest developments in new methods and analysis across European countries. This study was carried out under the InfAct (Information for Action) project [12] which is a joint action of Member States aiming to develop a more sustainable EU health information system through improving the availability of comparable, robust and policy-relevant health status data and health system performance information. InfAct gathers 40 national health authorities from 28 Member States (MSs). This study is part of a work package (WP9) focused on innovation in health information system (i.e., using data linkages and/or AI) to improve public health surveillance and health system performance for health policy development. The main objectives of this study were 1. to describe the current use of data linkage at the individual level and AI techniques applied in routine public health activities, 2. to identify the relevant health indicators (i.e., outcome and intervention indicators) estimated and health determinants of non-communicable diseases and 3. to identify that what are the obstacles to linking different data sources in routine public health surveillance and research.

## Methodology

We performed the following steps to achieve the objectives of this study:

### Literature search

We reviewed the existing literature published on the use of data linkage and AI (i.e., one technique of AI is machine learning) for health status monitoring using PubMed on Dec. 1, 2018. We included in our search peer-reviewed articles, systematic reviews and published reports in English language. The search strategies are reported in additional file 1. Based on this review, we identified different data sources used for data linkage, the use of artificial intelligence [AI]), health outcome and intervention indicators and determinants of health (Additional file 2). This was not an exhaustive search and it was performed only to identify any pre-existing questionnaire or relevant information that could be used for the development of our survey on the current practices in innovative use of data sources across European countries.

### Definition of innovative use of data sources

We developed the definition of “innovative use of data sources” in the context of public health and health information system activities and defined as:

- *The linkage of different data sources* (health surveys and/or disease-specific or population-based registries and/or national cohort and/or clinical research datasets and/or administrative data and/or electronic health records and/or X-data sources i.e., information on determinants of health and can include data on various exposures [Additional file 2]) with each other using linkage technology and/or
- *The use of AI* either applied to linked data or to an individual data set,

allowing a better understanding of what determines population health or to promote the efficiency of the health system and guide decision making at different geographical levels, or at another categorization parameter level.

### Development of the web-based survey

We developed a questionnaire and requested information from European countries on the data sources used for linkage, general characteristics of the data linkage, use of AI to estimate health indicators, related health outcomes and intervention indicators estimated and health determinants of non-communicable diseases. We reported these results according to three levels of using data linkage or AI in routine public health activities across European countries: *1. Advanced* (i.e., those who use data linkage or AI in routine to estimate health indicators), *2. In Progress* (i.e., those for whom the deployment of these innovative techniques [i.e., data linkage or AI] is still underway and expect to integrate these techniques in the next 5 years), and *3. Not yet* (i.e., those for which the use of these techniques is not foreseen yet).

Survey participants were also asked to report at least three health indicators, which are related to priority medical conditions in their country. We adopted the Euro-REACH Framework (i.e., it is a project based on an international collaboration to improve access to health care data through cross-country comparisons) [13] to classify the identified health outcome indicators, determinants of health and intervention indicators under the following categories: health outcome indicators (1. Health characteristics, 2. Mortality, 3. Human function and quality of life and 4. Life expectancy and well-being), determinants of health (1. Physical environment, 2. Socioeconomic and environment, 3. Health behavior and life style and 4. Biological /metabolic parameters) and intervention indicators (1. Prevention, 2. Promotion and 3. Others). We also asked specific information on the objective of estimation of health indicators (i.e., for public health monitoring, scientific research [clinical, epidemiology, public health], both), status of their use (i.e., was used, currently in use or could be produced in future) and level of estimation (i.e., national, sub-national, metropolitan, at all levels). If the same health indicator was reported more than once either as being estimated currently or to be estimated in future by different countries, we counted those health indicators once. The web-based questionnaire was developed using the Lime Survey tool by the Data lab of Santé Publique France. The questionnaire included both closed and open-ended questions (i.e., 20 questions). This questionnaire was reviewed by a group of experts on health information systems in their country and was revised according to their feedback before the launch of the survey. The web-based version of the questionnaire was pretested by the co-authors (SME, RH and RG) from the national public health institutes of Austria, France and Lithuania respectively, to check the clarity of the questions and contents.

The survey participants were the partners of the InfAct project and/or national representatives, experts, health information advisors in their countries, employed by their government, national institutes of public health, health information and statistics institutes or research departments of the universities.

The invitation email with an electronic link to the questionnaire was sent on April 1, 2019, to the identified representatives in 31 European countries [28 EU-MSs + 2 EEA (Iceland and Norway) + Others (Serbia)] and they were asked to complete the survey in four weeks (i.e., April 30, 2019)*.* For the United Kingdom, data were provided separately by three countries England, Scotland and Wales but counted as one member state. The first reminder was sent via-email one month after the survey launch, on May 3, 2019, and the second reminder another two weeks later, on May 23, 2019. The abbreviations of the member countries and the names of the survey respondents are reported in the additional file 3.

### Study outcomes

The main outcomes of this study were the current data linkage and AI practices and related health indicators estimated in routine public health activities across European countries. A descriptive analysis of the web-based questionnaire results was performed using Microsoft Excel.

## Results

### Literature search

**Fig. 1:**
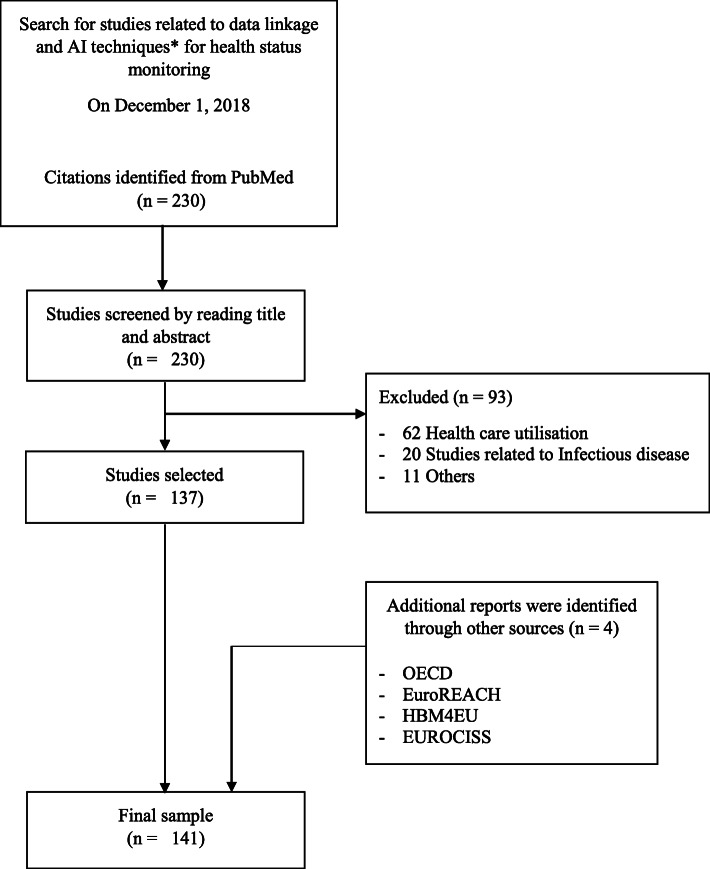
Flow diagram of studies using linked data and artificial intelligence for health status monitoring to develop a survey on identifying various data linkage practices across European countries in 2019. *To be more specific for AI techniques, we looked for studies using machine learning techniques (i.e., one type of AI technique) more often used for health status monitoring

We reviewed 137 citations from PubMed and four reports from the following organizations: OECD (Organization for Economic Co-operation and Development) [14], Euro-REACH [15], HBM4EU (Human Biomonitoring for Europe) [16], EUROCISS (European Cardiovascular Indicators Surveillance Set) [17], to develop this questionnaire (Fig. 1).

### Survey results

The survey results include the countries response, use of data linkage in routine public health activities, use of AI in routine public health activities, health indicators estimated using linked data and main obstacles to linking different data sources. All survey respondents have validated these results.

i. **Countries response**

Twenty-nine countries (i.e., EU MSs 27 + EEA 1 [Norway] + Others 1 [Serbia]) participated in the survey with a response rate of 94% (29/31). Hungary, Iceland and Northern Ireland did not participate.

ii. **Use of data linkage in routine public health activities**

Our survey results highlight that 24 European countries perform data linkage in their routine public health activities. These countries link administrative data such as EHRs (Electronic Health Records), mortality data, and disease specific registries whereas six of them (Cyprus, Italy, Poland, Portugal, Spain and Slovakia) are also developing this technique further to link with other types of data sources (i.e., demographic data, domestic/leisure accidents data, congenital anomalies registry). Ireland and Latvia have ongoing initiatives of data linkage (Table 1).

**Table 1:** Use of data linkage for routine public health activities in European countries in 2019

Use of Data Linkage			
	Advanced N = 24	In progress* N = 8	Not yet N = 3
**European Countries**	AT, BE, BG, CY, CZ, DE, DK, EE, ES, FI, FR, HR, IT, LT, MT, NL, NO, PL, PT, SI, SK, SRB, SW, UK (ENG, SC, WL)	CY, ES, IE, IT, PL, PT, SK, LV	GR, LU, RO

* 6 countries (CY, ES, IT, PL, PT & SK) use data linkage in routine (i.e., advanced) but also developing further this technology to link different other data sources (i.e., in progress)

Three countries (Greece, Luxembourg and Romania) have not yet planned to integrate data linkage in their routine public health activities. The following reasons were mentioned by some countries for not having institutionalized data linkage: lack of a public health institution, which should collect and govern the health related data, data linkage is not part of the health agenda, lack of commitment from the ministry of health, lack of resources to establish a national health information system, the institutional complexity of the Ministry of Health and strict laws and regulations, which hinder data linkage with different data sources.

#### Objectives of data linkage

Data linkage can be performed in routine for different objectives such as for health status monitoring, health system performance, health policy development or for scientific research (i.e., public health, epidemiology or clinical) purposes. Our results showed that data linkage was performed for health status monitoring in 20 countries (BE, CY, CZ, DE, DK, EE, ES, FI, FR, HR, IT, LT, MT, NL, PT, SI, SK, SRB, SW, UK (SC, WL), for health policy development in 13 (AT, BE, BG, DK, EE, FR, MT, NL, NO, PL, SK, SW, UK (SC, WL) and for scientific research (public health, epidemiological and clinical) purposes in 13 (BE, CZ, DE, DK, EE, ES, FI, FR, NL, PT, SI, SW, UK (ENG, SC, WL). Finland, Spain, Sweden and Scotland also perform data linkages to identify population risk factors. In Sweden, data linkage is also used to monitor compliance with national treatment guidelines to improve health care quality.

#### Data sources used for linkage

Our results showed that 24 European countries, who perform data linkage in routine, use most frequently the five following types of data sources: health-related administrative data sources, non-health related administrative data sources, disease-specific registries, national health surveys, population-based epidemiological cohort and clinical trials (Additional file 4). These data sources are linked with each other in different combinations and some examples of the various combinations used across member countries, are reported in additional file 5. These countries perform data linkage based on the following information: social security number, patient unique identification number, person unique pseudonymous identifier, encrypted personal identification number, citizen or national identification number. In some countries, for instance in Ireland, the lack of unique patient identifier number, limits the potential to link with different data sources.

#### General characteristics of linked dataset

Our results showed that among 24 European countries who perform data linkage in routine, 17 do linkage at national level (Table 2). France, Portugal and Scotland do data linkage both at national and sub-national levels. Denmark, Germany, Norway and Sweden do data linkage at all levels. 23 countries either perform the deterministic type of linkage (12 countries) or a combination of deterministic and probabilistic linkage (11 countries). In 16 out of 24 countries, linked data is available and is used in routine. In 12 out of 24 countries, the register owner (i.e., who governs the data register) provides the approval to access linked data. In 15 out of 24 countries, the accessibility to linked data is in routine or permanent whereas, in 13 countries, the accessibility could be ad-hoc or at intermittent basis depending on the project. In 15 out of 24 countries, linked data do not operate in real-time (i.e., integrate the updated information with minimum delay in time). In 19 out of 24 countries, linked data are flexible to integrate new variables.

**Table 2:** General characteristics of linked datasets in European countries in 2019

S/No	General characteristics of linked datasets	European countries		
		Advanced N = 24		In progress N = 2
**1**	**Level of data linkage use/implementation**			
	National level	17	AT, BE, BG, CY, CZ, EE, ES, FI, HR, LT, MT, NL, NO, SI, SK, SRB, UK (ENG, WL)	IE, LV
	Sub-national level	1	IT	
	Both (National and Sub-national) levels	3	FR, PT, UK-SC	
	Metropolitan level	4	MT, PL, SI, UK-WL	
	All of above	4	DE, DK, NO, SW	
**2**	**Type of linkage**			
	Deterministic	12	AT, CY, HR, FI, LT, MT, NL, NO, SI, SK, SRB, SW	IE, LV
	Probabilistic	1	UK-SC	
	Combination of both (i.e., deterministic and probabilistic)	11	BE, CZ, DE, DK, EE, ES, FR, IT, PL, PT, UK (ENG, WL)	
	None of the above	1	BG	
**3**	**Current status of linked data usage**			
	Available and is used in routine	16	AT, BE, CY, CZ, DK, EE, FI, FR, LT, MT, NL, NO, PL, SI, SW, UK (ENG, SC, WL)	
	In progress of development	4	BG, ES, HR, PT	IE, LV
	Partial in use & partial in progress of development	2	DE, SK	
	Available but not in use	2	IT, SRB	
**4**	**Type of approval to access**			
	By law	5	AT, CZ, MT, NO, SW	
	By ethical committee	7	BE, ES, FR, IT, NO, PT, UK (ENG, SC, WL)	
	By register owner	13	BG, CY, ES, HR, FI, FR, IT, NO, PL, PT, SI, SK, SRB	
	Others (i.e., depend on linkage/data protection inspector/under conditions/not applicable (data linkage in safe environment)/by statistical authority	7	CZ, DE, DK, EE, ES, LT, NL	LV
**5**	**Type of accessibility**			
	In routine/permanent	15	BE, BG, CZ, DK, EE, FI, FR, NL, NO, PL, PT, SI, SK, SW, UK (ENG, SC, WL)	
	Ad-hoc/Intermittent	13	BE, CY, DE, EE, ES, HR, IT, FR, MT, NO, PT, SK, SRB	
	Under conditions (i.e., restricted to certain projects for a limited period)	6	AT, EE, ES, FR, LT, NO	LV
**6**	**Operate in real-time**			
	Yes	10	DK, EE, FI, FR, LT, NO, SI, SK, SW, UK-SC	
	No	15	AT, BE, BG, CY, CZ, DE, ES, HR, IT, MT, NL, PL, PT, SRB, UK (ENG, WL)	IE, LV
**7**	**Flexible to integrate new variables**			
	Yes	19	AT, BG, CY, CZ, DE, DK, ES, FI, FR, HR, MT, NL, NO, PL, PT, SI, SK, SW, UK (ENG, SC, WL)	
	No	5	BE, EE, IT, LT, SRB	IE, LV

There are ongoing projects to integrate data linkage (i.e., in next five years) as part of this technology in their routine public health activities in following European countries: Austria, Cyprus, Czech Republic, Ireland, Italy, Latvia, Norway, Poland, Portugal, and Spain.

iii. **Use of artificial intelligence (AI) in routine public health activities**

The use of AI is not frequent across European countries (Table 3). Only five countries have reported applying the following techniques in routine public health activities: machine learning (Denmark, Finland, Sweden, and UK-Wales), natural language processing (Finland, Sweden, and UK-Wales), Markov decision process (Finland), support vector machine (Finland, UK-Wales), data mining (Finland) and TSP [Travelling Salesman Problem] modelling (Norway). Denmark can apply these techniques not only at national level but also at metropolitan level.

**Table 3:** Use of artificial intelligence in routine public health activities in European countries in 2019

Use of Artificial Intelligence (AI)			
	Advanced N = 5	In progress N = 9	Not yet N = 16
**European countries**	DK, FI, NO, SW, UK-WL	AT, CZ, DE, ES, FR, HR, PL, PT, SK	BE, BG, CY, EE, GR, IE, IT, LT, LU, LV, MT, NL, RO, SL, SRB, UK (ENG, SC)
**Level of application of AI**			
National level	DK, FI, NO, SW, UK- WL		
Sub-national level			
Metropolitan level	DK, SW		
**Use of classical statistics without the use of AI**			
	**Advanced** **N = 19**	**In progress** N = 5*	**Not yet** **N = 8**
**European countries**	BE, BG, CZ, DE, EE, ES, DK, FR, FI, IT, MT, NL, NO, PL, PT, SI, SK, SW, UK (ENG, SC, WL)	AT, CZ, ES, HR, SK	CY, GR, IE, LT, LU, LV, RO, SRB
**Level of use of classical statistics without AI**			
National level	BE, BG, CZ, DK, EE, FR, FI, IT, NL, NO, PL, PT, SK, SW, UK- WL		
Sub-national level	DE, ES, IT, PL, NO, SI, UK (ENG, SC)		
Metropolitan level	DK, MT, NO		

*Two countries (CZ & SK) use classical statistic in routine (i.e.*, advanced) but also developing further this technology (*i.e.*, in progress)*

There are ongoing projects to integrate the use of AI in routine public health activities in the next five years in following countries: Croatia, Czech Republic, France, Germany, Norway, Portugal, and Spain. The objectives of these initiatives are for epidemiological research and surveillance of non-communicable and communicable disease estimating the prevalence and prediction of incidences of certain health conditions at various geographical levels.

Two countries mentioned that due to lack of human resources (Lithuania) and capacities/skills (Republic of Serbia) within their public health institutes, AI techniques are not applied in routine public health activities.

Some European countries also mentioned the use of classical statistical techniques without the use of AI (Table 3).

iv. **Health indicators estimated using linked data**

Using linked data, the majority of European countries estimate the following health indicators:

#### Health outcome indicators

Participants were asked to select at least three health conditions and to report the related health outcome indicators, which are most important for public health in their country. Using linked data, 46 health outcome indicators related to the following seven health conditions were reported from 22 countries: c*ardiovascular* (14), neurodegenerative disease (6), maternal and perinatal health (6), diabetes (6), suicide/trauma/injury (7), cancer (6) and hepatic failure (1) (Additional file 6). The main objectives to estimate these indicators were for public health monitoring and research purposes. The level of estimation was mainly at national and sub-national levels. These 46 health outcome indicators were classified according to the following categories: 1. health characteristics, 2. mortality, 3. human function and quality of life and 4. life expectancy and well-being. For example for the first category, Czech Republic, France, Lithuania, Sweden and Wales, use linked data in routine public health surveillance to estimate the incidence and prevalence of diabetes (Additional file 6).

#### Health determinants

Participants were also asked to report the corresponding determinants of the identified health conditions. 34 health determinants related to various health conditions were reported by 15 member states *(*Table 3*.2).* These determinants are related to the physical environment (12), socioeconomic status and the environment (10), health behavior and lifestyle (6) and biological and metabolic parameters (3) (Additional file 7). These determinants were used to measure the potential associations between these risk factors and health conditions for public health monitoring and research purposes. These determinants can be stratified by age, sex, socioeconomic status and by area of residence. For example in England and Wales, in relation to the physical environment, the proximity of fast food outlets from areas of residence is used to measure its potential association with chronic health conditions such as adiposity and obesity. This variable can be stratified by the area of residence (Additional file 7).

#### Health intervention indicators

Participants were asked to report at least three health intervention indicators under three categories (i.e., prevention, promotion, others) corresponding to the given health conditions which are most important for public health in their country. Using linked data, 23 health intervention indicators related to the following six health conditions were reported from 17 member states: maternal and perinatal health (7), cancer (6), diabetes (4), cardiovascular (2), neurodegenerative disease (2), suicide/trauma/injury (1) and lower/upper respiratory infections (1), (Additional file 8). The main objectives to estimate these indicators were to guide the health policy process, public health monitoring and for research. These intervention indicators are estimated mainly at national and sub-national levels and currently are in use. For example in Sweden, one of the estimated intervention indicator relates to preventive therapy, the number of diabetic patients counselled by a nurse to avoid complications (Additional file 8).

v. **Main obstacles to linking different data sources**

The majority of European countries we surveyed identified the following main obstacles associated with the implementation and the use of data linkage and advanced statistics: 1. The complex laws and data protection regulations, which block linkage between different data sources with a deterministic approach *(legal)*, 2. Lack of human resources and capacities/skills within national institutes of public health and health information statistics *(technical)*, 3. Lack of governance of health information *(data governance)* and 4. Limited resources to support the health information infrastructure *(organization and structural)*.

## Discussion

The results of this study showed the variability in the use of data linkage and AI at national institutes of public health, health information and statistics across European countries. The majority of countries use data linkage in routine by applying either deterministic or a combination of two types of linkages (i.e., deterministic & probabilistic) for public health surveillance and research purposes. The use of a universal unique identifier, social security number or unique pseudonymous identifier is common to applying deterministic linkage technique among European countries. The use of AI is less common to estimate health indictors at national public health institutes. Across European countries, using data linkage, 46 health outcome indicators related to seven health conditions, 34 related to determinants and 23 health intervention indicators were reported. Some initiatives are ongoing as pilot projects to test these techniques to improve health surveillance and to guide health policy development. Four main obstacles to linking different data sources have been identified by the European experts.

A systematic review has shown that data linkage is used in the field of perinatal health for both health surveillance and research purposes in European countries [8]. Several other studies have shown that linkage is used to explore various dynamics of population health such as social care, psychotic disorders, multi-morbidity, diabetes, obesity, mental health, cardiovascular, antibiotic use and Alzheimer using data linkage with different types of administrative data sources (both related to health and non-health) [7, 18–30]. For the surveillance of cancer, data linkage not only provides the opportunity to improve the population-based screening [31] but it also helps to detect different types of cancer recurrence [32] and to evaluate the socio-economic status of patients with cancer (e.g., return to work) [33]. Linked data also allows evaluating the health interventions at various levels of the population [34]. The diversity and the volume of health information have been increasing rapidly and push to discover new parameters to improve population health with innovative approaches. In that context, some initiatives have been launched at national level to create health data hub/platform to be used for research and to guide the policy development process [35, 36].

As for AI, there are some studies available which have discussed the advantages of using this technology for early detection and diagnosis of certain medical conditions, for treatment, as well as for outcome prediction and prognosis evaluation with a high level of precision [11, 37] but the use of AI at population level to estimate and predict health indicators remains limited [38]. Furthermore, this technique also permits exploring underlying unobserved trends and patterns in large datasets without priori hypothesis.

Our results highlighted that a few member countries have achieved the most advanced levels of data linkage by linking health information (i.e., clinical, biobanks/laboratory tests, genetics) with non-health population data sources and registers of education, occupation, housing quality, air pollution, criminal statistics and transport/road accidents, etc. These innovative linkages offer the exceptional opportunities to enrich information, investigate non-health determinants and to perform high quality epidemiological research, health surveillance, to guide health policies to improve population health. These innovative linkages are especially relevant and of high value when considering health-in-all policies approaches. However, the majority of European countries have not reached that level of use in data linkage and AI and underlined main obstacles associated with the implementation and the use of data linkage and AI. These are related to legal, technical and data governance issues as well as organizational and structural aspects.

To address these issues and to increase the uptake of innovative and high-performance technologies in public health activities, we propose the following recommendations*: A. Legal aspects:* 1. More flexible data governance frameworks to support data linkage of different data sources should be encouraged [39], 2. Specific mandates to ensure data availability/access/capture and safe storage should be an integral part of a national/regional health information system, 3. Differences in the implementation and interpretation of the EU-GDPR (General Data Protection Regulations) and additional national regulations should be mapped and if possible harmonized across EU-MSs [40]; *B. Technical aspects:* 4. More collaborations and partnerships should be encouraged to build up capacities for using new health information related technologies, to share new methods, skills, experiences and data for comparative research studies among EU national institutes of public health, health information and statistics; *C. Data  governance*, 5. Initiatives to strengthen national health information infrastructure should be encouraged; *D. Organizational and structural aspects*, 6. Ministries of health and research from European countries should provide their support (i.e., financial and political) for the development of integrated national health data hubs/data platforms to strengthen the national health information infrastructure.

There are a few limitations in this study. *First,* our study may not have a complete coverage of data linkage practices within countries. The current linkages reported by national institutes of public health, health information and statistics we surveyed may differ from those conducted by other research institutes in that country which we did not cover in the survey. This might influence the results of this study. *Second,* we acknowledge that there are other innovative methods exist such as use of data clouds or blockchain. However, these methods are not frequently used for routine public health surveillance and research purposes. *Third,* we limited the response burden of health indicators to three priority health conditions. Therefore, our results do not constitute an exhaustive list of health indicators that are used in the country to inform policy and practice. It may limit the number of health indicators being estimated using linked data and advanced statistics. Nevertheless, this survey provides the latest overview of current practices in data linkage and AI in European countries and highlights the related obstacles in using these technologies for routine public health activities.

## Conclusions

To our knowledge, this is the first study, which provides the information on current practices of data linkage and AI use at national institutes of public health, health information and statistics across European countries. Our results highlight that the majority of countries have integrated data linkage in their routine public health activities but few use AI. The European countries who are advanced in using both techniques i.e., data linkage and AI, could guide others by an exchange of their experiences and examples of good practices. A sustainable national health information system and flexible data governance frameworks to link different data sources are essential to support evidence-based practices for health policy development. Building analytical capacity and raising awareness of the added value of data linkage in national institutes of public health, health information and statistics is necessary for increasing the use of linked data in order to improve the quality of public health surveillance and monitoring activities. The recommendations we have put forth could ultimately contribute to strengthening national health information systems in Europe and would facilitate moving towards the establishment of an integrated EU- Health Information System.

## Supplementary information

**Additional file 1.** It is a doc. Word file. It describes the search strategies used to identify citations related to data linkage and machine learning technique used for health status monitoring.

**Additional file 2.** It is a doc. Word file. It describes the definitions of different data sources used for data linkage for health surveillance and research purposes.

**Additional file 3.** It is a doc. Word file and include a table describing the names of survey respondents, their institutes and email addresses.

**Additional file 4.** It is a doc. Word file and describes different data sources used for linkage across European countries in 2019.

**Additional file 5.** It is a doc word file and describes examples of different combinations of data linkage across European countries in 2019.

**Additional file 6.** It is a doc. Word file and describes examples of health outcome indicators estimated using linked data across European countries in 2019.

**Additional file 7.** It is a doc. Word file and describes examples of health determinants identified using linked data across European countries in 2019.

**Additional file 8.** It is a doc. Word file and describes examples of health intervention indicators estimated using linked data across European countries in 2019.

## Data Availability

Not applicable.

## Abbreviations

AI: Artificial Intelligence
EU: European Union
EEA: European Economic Area
MSs: Member States
InfAct: Information for Action i.e., a joint action of Member States to establish a sustainable European health information system
WP: Work Package
Euro-REACH: It is an international collaboration to improve access to health care data through cross-country comparisons
OECD: Organization for Economic Co-operation and Development
HBM4EU: Human Biomonitoring for Europe
EUROCISS: European Cardiovascular Indicators Surveillance Set
PCA: Principal Component Analysis
GDPR: General Data Protection Regulations
