# Supplementary material for: Innovative use of data sources: a cross-sectional study of data linkage and artificial intelligence practices across European countries
Source: Arch Public Health. 2020 Jun 10;78:55. doi: 10.1186/s13690-020-00436-9 (PMC7288525; doi:10.1186/s13690-020-00436-9)
Supplement: Supplementary file 1 — Additional file 1. It is a doc. Word file. It describes the search strategies used to identify citations related to data linkage and machine learning technique used for health status monitoring. [file 13690_2020_436_MOESM1_ESM.docx]

**Additional file 1: Search strategies**

Search strategy 1

((Health status monitoring [Title/Abstract] OR Surveillance [Title/Abstract]) AND Linked data [Title/Abstract]))

Search strategy 2

((Health status monitoring [Title/Abstract]) OR Surveillance [Title/Abstract]) AND Machine learning approach [Title/Abstract]))
