# Supplementary material for: Innovative use of data sources: a cross-sectional study of data linkage and artificial intelligence practices across European countries
Source: Arch Public Health. 2020 Jun 10;78:55. doi: 10.1186/s13690-020-00436-9 (PMC7288525; doi:10.1186/s13690-020-00436-9)
Supplement: Supplementary file 2 — Additional file 2. It is a doc. Word file. It describes the definitions of different data sources used for data linkage for health surveillance and research purposes. [file 13690_2020_436_MOESM2_ESM.docx]

**Additional file 2: Definitions of data sources**

Here we describe the definitions of different types of data sources, artificial intelligence techniques applied, health outcome, determinants and intervention indicators.

**Different types of data sources:**

1. ***Health surveys/Population health surveys*** collect information of risk factors, health behaviors and non-health care determinants of health^1^. Health surveys are used to measure the prevalence of risk factors and healthy behavior, monitor the effects of interventions, measure community attitudes to health policy initiatives, as well as assess trends in health and disease outcomes. Health surveys could involve health interview surveys or health examination surveys. These health surveys will include those surveys which are performed either at national or sub-national levels.
2. ***Disease-specific or population-based registries:*** A registry is a collection of information about individuals, usually focused around a specific diagnosis or condition^2^. Many registries collect information about people who have a specific disease or condition, while others seek participants of varying health status who may be willing to participate in research about a particular disease. Individuals provide information about themselves to these registries on a voluntary basis. Registries could be disease-specific registries, screening registries, immunization registries, etc.
3. ***National cohorts*** are performed to investigate the causes of development of major chronic diseases, i.e. cardiovascular diseases, cancer, diabetes, neurodegenerative/-psychiatric diseases, musculoskeletal diseases, respiratory and infectious diseases, and their pre-clinical stages or functional health impairments at the national level.
4. ***Clinical trials data*** include data on safety and efficacy of interventions. This data may be available through national or international trial registries. For example, ClinicalTrials.gov, Cochrane Library, WHO International Clinical Trials Registry Platform (ICTRP), European Union Clinical Trials Database, etc.
5. ***Administrative data*** ***sources*** were initially developed for administrative use, not for public health surveillance and have a larger coverage of population. For example, birth certificates, death certificates, census, biobank data (i.e., it is a biorepository that accepts, processes, stores and distributes bio specimens [i.e., blood, urine, spinal fluid, etc.] and associated data for use in research and clinical care^3^), GIS (Geographical Information System/GPS/Geodata), socioeconomic data, and retirement/pension data, etc.
6. ***Electronic health/patients/medical records (EHRs)*** include a summary of administrative data, clinical data of patients and determinants of health indicators (i.e., various types of exposures). In scientific literature, electronic health records (EHRs) are often refer to patients’ record or electronic medical records. These data sources are flexible to link with different types of data sources.

“EHRs are described as a repository of patient data in digital form^4^and include the following information: active and past diagnosis; past medical history; physical examinations; laboratory test orders and results; current prescriptions; radiological images and reports; hospitalization information; consultant reports; details of emergency care; immunizations; pathology reports; social history; lifestyle; allergies; genetic information; health screening study results; physicians, nurse, social worker, physical therapy notes at admission and discharge^5^.”

EHRs may include the following data sources:

1. *Hospital Record (HR)/In hospital medical record* includes information about a patient generated during a period of hospitalization with written accounts of consultants’ opinions as well as nurses’ observations and treatments^6^.
2. *Hospital Discharge Record is* a clinical report prepared by a physician or other health professional that summarizes the patient's chief complaint, the diagnostic findings, the therapy administered and the patients' response to it, and recommendations on discharge^7^.
3. *General Practitioner (GPs)/Primary care* include information on diagnoses and symptoms, laboratory test results, referrals to specialists and drug or healthcare product prescribed^8^.
4. *Specialist care* (i.e., cardiologists, neurologists, gynecologists, etc.) includes a highly skilled in a specific and restricted medical field^9^.
5. *Emergency care (i.e., emergency room and outpatient emergency)* include information on evaluation and initial treatment of medical conditions caused by trauma or sudden illness^10^.
6. *Health insurance claim (i.e., healthcare reimbursement)* is a detailed invoice that a health care provider (such as doctor, clinic, or hospital) sends to the health insurer to reimburse the expenses spent on health services (i.e., drugs, diagnostic/laboratory tests, etc.) received by a patient^1^.
7. *Drug prescription* include information on prescription date, type of drug, strength, dosage regimen, quantity, and route of administration^11^.
8. *Genomic/DNA data* sources (i.e., information about functions of specific genes and to assess the association of gene mutations in certain diseases ^2^ such as for breast cancer BRCA 1/2).
9. ***X-data sources)*:** These type of data sources provide precise information on determinants of health and can include data on various exposures such as biological parameters, social behavior, life style, physical environment, nutrition, etc. These sources are considered as part of big data (i.e., voluminous amount of [structured](https://whatis.techtarget.com/definition/structured-data), [semi structured](https://whatis.techtarget.com/definition/semi-structured-data) and [unstructured](https://searchbusinessanalytics.techtarget.com/definition/unstructured-data) data that has the potential to be mined for information ^12^).

We grouped these types of data sources as “X-data sources”. Some of these data sources are enlisted below and more could be possible:

1. *m-Health (mobile-Health)* is the use of mobile phones, wireless health apps and wearable devices and measure a set of biological parameters which could be used for disease surveillance and health care services^13^. Data from these apps can also be used for disease surveillance, treatment support, epidemic outbreak tracking and chronic disease management^13^.
2. *Social media* is an electronic communication through which users create online communities to share information, ideas, personal messages, and other content (such as videos) and the related data may reflect user’s social behavior towards different aspects^14^.
3. *Mobility mode data* (i.e., commuting for work by walking, using bicycle, public transport such as metro, train, bus, etc.) provide information about the mobility mode of general population whether walking or using different means of transport.
4. *Build physical environment* include data regarding green spaces, environmental exposure in terms of cleanliness, sound pollution, air quality, etc.
5. *Nutrition:* direct producers of seasonal/local fruits and vegetables, etc.
6. *Housing infrastructure* data source provide information about housing space and location.

**Artificial intelligence (AI) techniques**

The artificial intelligence techniques following techniques: machine learning, natural language processing, markov decision process, support vector machine, data mining, regression, etc., to analyze, estimate and predict the health indicators either from linked data or using an individual data set.

**Artificial Intelligence**

Markov decision process

Natural language processing

Support vector machine

Data mining

Others

Machine learning

**Health outcome and intervention related indicators and determinants of health**

We have selected the following non-communicable diseases based on burden of disease with higher incidence of mortality and morbidity across EU member states^15^: cardiovascular diseases, cancer, chronic obstructive pulmonary diseases, diabetes, neurodegenerative disease, mental health, accidents/trauma, maternal and perinatal health and any other disease.

Health outcomes indicators which are estimated from linked data and/or by using AI techniques to an individual data set, will be identified.

We have adopted EuroREACH Framework describing health status monitoring to classify the identified health outcome indicators, non-healthcare determinants of health and health intervention indicators under different categories^11^ *(see figure 2).*

**Health Status**

How healthy are citizens of member state, what their health outcomes are and what are their need for health care?

**Health Outcome Indicators**

Life Expectancy and well-Being

Human Function and Quality of Life

Health Characteristics

Mortality

**Determinants of Health**

What are the factors that determine health?

Biological/metabolic parameters

Health Behaviors and Lifestyle

Physical Environment

Socio-Economic Conditions and Environment

**Health Intervention Indicators**

Prevention indicator

Promotion indicator

Others

Figure 2: EuroREACH Framework for health status monitoring

***Health outcome indicators*** which are estimated either from linked data or by applying AI techniques to an individual data set, describe the health status of a population in terms of health characteristics (e.g., prevalence of stroke among ≥ 65 years old), human function and quality of life (e.g., quality of life after stroke), life expectancy and well-being (e.g., survival of people with stroke) and mortality (e.g., causes of mortality).

These outcome indicators have the potential to improve health surveillance with more precise information. These health outcome indicators include prevalence, incidence, population attributable risk, population attributable fraction, relative risk, hazard ratio, etc.

***Determinants of health*** which are identified either from linked data or from an individual data set, can provide more variables/parameters to better understand exposures factors related to health behavior and lifestyle (i.e., risk and/or protective behavior, response to health problems, etc.), biological/metabolic parameters (i.e., genetic, body structure and functioning, etc.), socio-economic conditions and environment (i.e., attitudes, social networks, education, employment, living standard, etc.) and physical environment (i.e., water quality, air quality, food safety, etc.). For example, use of public transport to commute for work.

These determinants should be different from already compiled databases of OECD, WHO or Eurostat.

***Health intervention indicators*** which are estimated either from linked data or by applying AI techniques to an individual data set, describe the effect of the interventions applied in terms of prevention (e.g., the use of genetic screening of BRCA1/2 genes among families with a family history of breast cancer) or promotion (i.e., integrated health programs at work place, schools, hospitals, policies and practices on healthy lifestyle, etc.).

**References:**

1. Madans JH. Health Surveys. In: Smelser NJ, Baltes PB, eds. *International Encyclopedia of the Social & Behavioral Sciences*. Oxford: Pergamon; 2001:6619-6627.

2. Health NIo. What is a registry? <https://www.nih.gov/health-information/nih-clinical-research-trials-you/list-registries>. 2018.

3. De Souza YG, Greenspan JS. Biobanking Past, Present and Future: Responsibilities and Benefits. *AIDS (London, England).* 2013;27(3):303-312.

4. 20514 IT. Health Informatics-Electronic Health Record-Definition, Scope, and Context: <https://www.iso.org/obp/ui/#iso:std:iso:tr:20514:ed-1:v1:en>. 2005.

5. Häyrinen K, Saranto K, Nykänen P. Definition, structure, content, use and impacts of electronic health records: A review of the research literature. *International Journal of Medical Informatics.* 2008;77(5):291-304.

6. Katzenellenbogen JM BS, Somerford P, Anderson CS, Semmens JB, Codde JP, Vos T. Disability burden due to stroke in Western Australia: new insights from linked. *Int J Stroke.* 2010;5(4):269-277.

7. White C GM, Johnson B, Corbin T. Social inequalities in adult male mortality by the National Statistics. *Health Stat Q.* 2007;36:6-23.

8. Manuel DG SS. Health-related quality of life and health-adjusted life expectancy of people with. *Diabetes Care.* 2004;27(2):407-414.

9. Tuppin P, Rudant J, Constantinou P, et al. Value of a national administrative database to guide public decisions: From the systeme national d'information interregimes de l'Assurance Maladie (SNIIRAM) to the systeme national des donnees de sante (SNDS) in France. (0398-7620 (Print)).

10. Dunnell K BJ, Wood R, Babb P. Measuring aspects of women's life and work for the study of variations in health. *Am J Ind Med.* 1999;36(1):25-33.

11. EuroREACH. EuroREACH Framework: <http://hdn.euhs-i.eu/performance/frameworks/euroreach-framework>. 2013.

12. Big data: <https://searchdatamanagement.techtarget.com/definition/big-data>.

13. Lloyd K, McGregor J, John A, et al. A national population-based e-cohort of people with psychosis (PsyCymru) linking prospectively ascertained phenotypically rich and genetic data to routinely collected records: Overview, recruitment and linkage. *Schizophrenia Research.* 2015;166(1):131-136.

14. Atramont A, Bonnet-Zamponi D, Bourdel-Marchasson I, Tangre I, Fagot-Campagna A, Tuppin P. Health status and drug use 1 year before and 1 year after skilled nursing home admission during the first quarter of 2013 in France: a study based on the French National Health Insurance Information System. *European Journal of Clinical Pharmacology.* 2018;74(1):109-118.

15. WHO. Global status report on noncommunicable diseases: <http://www.who.int/nmh/publications/ncd_report_full_en.pdf>. 2010.
