# Supplementary material for: Innovative use of data sources: a cross-sectional study of data linkage and artificial intelligence practices across European countries
Source: Arch Public Health. 2020 Jun 10;78:55. doi: 10.1186/s13690-020-00436-9 (PMC7288525; doi:10.1186/s13690-020-00436-9)
Supplement: Supplementary file 3 — Additional file 3. It is a doc. Word file and include a table describing the names of survey respondents, their institutes and email addresses. [file 13690_2020_436_MOESM3_ESM.docx]

**Additional file 3: Abbreviation of European Countries with contact detail of survey respondents**

| **S/No** | **Abbreviations** | **Country** | **First name** | **Last name** | **Institute** | **Email** |
| --- | --- | --- | --- | --- | --- | --- |
| 1 | AT | Austria | Stefan | Mathis-Edenhofer | Public Health Institute | stefan.mathis-edenhofer@goeg.at |
| 2 | BE | Belgium | Herman | Van Oyen | Sciensano | [herman.vanoyen@sciensano.be](mailto:herman.vanoyen@sciensano.be) |
| 3 | BG | Bulgaria | Raina | Nikolova | Public Health Institute | r.nikolova@ncpha.government.bg |
| 4 | HR | Croatia | Ivan | Pristas | CIPH (HZJZ) | [ivan.pristas@hzjz.hr](mailto:ivan.pristas@hzjz.hr) |
| 5 | CY | Cyprus | Vasilios | Scoutellas | Public Health Institute | [VScoutellas@mphs.moh.gov.cy](mailto:VScoutellas@mphs.moh.gov.cy) |
| 6 | CZ | Czech Republic | Sarka | Dankova | UZIS | sarka.dankova@uzis.cz |
| 7 | CZ | Czech Republic | Jiri | Jarkovsky | Institute of Health Information and Statistics | Jiri.Jarkovsky@uzis.cz |
| 8 | DK | Denmark | Mette Bjerrum | Koch | Danish Health Data Authority | [mebk@sundhedsdata.dk](mailto:mebk@sundhedsdata.dk) |
| 9 | DK | Denmark | Maja Bæksgaard | Jørgensen | Statens Institut for Folkesundhed | mbha@sdu.dk |
| 10 | EE | Estonia | Eleri | Lapp | MoSA | [eleri.lapp@sm.ee](mailto:eleri.lapp@sm.ee) |
| 11 | FI | Finland | Mika | Gissler | THL | [mika.gissler@thl.fi](mailto:mika.gissler@thl.fi) |
| 12 | DE | Germany | Angelika | Schaffrath Rosario | RKI | Schaffrath-RosarioA@rki.de |
| 13 | EL | Greece | Spyridon | Goulas | Governmental Health Insurance Organization | [sgoulas@eopyy.gov.gr](mailto:sgoulas@eopyy.gov.gr) |
| 14 | FR | France | Anne | Gallay | Public Health Institute | [Anne.GALLAY@santepubliquefrance.fr](mailto:Anne.GALLAY@santepubliquefrance.fr) |
| 15 | FR | France | Jennifer | Zeitlin | INSERM U 1153 –Research Institute | [jennifer.zeitlin@inserm.fr](mailto:jennifer.zeitlin@inserm.fr) |
| 16 | IE | Ireland | Sheona | Gilsenan | DOH | [sheona_gilsenan@health.gov.ie](mailto:sheona_gilsenan@health.gov.ie) |
| 17 | IT | Italy | Luigi | Palmieri | ISS | [luigi.palmieri@iss.it](mailto:luigi.palmieri@iss.it) |
| 18 | IT | Italy | Brigid | Unim | ISS | brigid.unim@iss.it |
| 19 | LV | Latvia | Janis | Misins | CDPC | [janis.misins@spkc.gov.lv](mailto:janis.misins@spkc.gov.lv) |
| 20 | LT | Lithuania | Rita | Gaidelyte | HI | rita.gaidelyte@hi.lt |
| 21 | LU | Luxembourg | Anne-Charlotte | Lorcy | Health-Directorate | [anne-charlotte.lorcy@ms.etat.lu](mailto:anne-charlotte.lorcy@ms.etat.lu) |
| 22 | MT | Malta | Neville | Calleja | MFH | [neville.calleja@gov.mt](mailto:neville.calleja@gov.mt) |
| 23 | NL | Netherlands | Peter | Achterberg | RIVM | [peter.achterberg@rivm.nl](mailto:peter.achterberg@rivm.nl) |
| 24 | NO | Norway | Hakon | Haaheim | National Health Directorate | [hakon.haaheim@helsedir.no](mailto:hakon.haaheim@helsedir.no) |
| 25 | PL | Poland | Jakub | Adamski | MoH | [j.adamski@mz.gov.pl](mailto:j.adamski@mz.gov.pl) |
| 26 | PL | Poland | Piotr | Nowosielski | MoH | [p.nowosielski@mz.gov.pl](mailto:p.nowosielski@mz.gov.pl) |
| 27 | PT | Portugal | Paulo | Nogueira | DGS | [paulo.nogueira@dgs.min-saude.pt](mailto:paulo.nogueira@dgs.min-saude.pt) |
| 29 | PT | Portugal | Martins | José | Public Health Institute | [josemartins@dgs.min-saude.pt](mailto:josemartins@dgs.min-saude.pt) |
| 30 | PT | Portugal | Carlos | Dias | Public Health Institute | [carlos.dias@insa.min-saude.pt](mailto:carlos.dias@insa.min-saude.pt) |
| 31 | RO | Romania | Silviu | Radulescu | Public Health Institute | [silviu.radulescu@insp.gov.ro](mailto:silviu.radulescu@insp.gov.ro) |
| 32 | RS | Serbia | Maja | Krstic | Batut | [maja_krstic@batut.org.rs](mailto:maja_krstic@batut.org.rs) |
| 33 | SK | Slovakia | Jan | Cap | National Health Information Centre | [Jan.Cap@nczisk.sk](mailto:Jan.Cap@nczisk.sk) |
| 34 | SI | Slovenia | Metka | Zaletel | NIJZ | [metka.zaletel@nijz.si](mailto:metka.zaletel@nijz.si) |
| 35 | ES | Spain | Beatriz | Perez-Gomez | ISCIII | [bperez@isciii.es](mailto:bperez@isciii.es) |
| 36 | SE | Sweden | Hanna | Lobosco | MoH | [hanna.lobosco@folkhalsomyndigheten.se](mailto:hanna.lobosco@folkhalsomyndigheten.se) |
|  | SE | Sweden | Rosita | Wigand | Public Health Agency of Sweden | rosita.wigand@folkhalsomyndigheten.se |
|  | SE | Sweden | Jenny | Borlin | Public Health Agency of Sweden | jenny.borlin@folkhalsomyndigheten.se |
| 37 | UK-ENG | England | Robert | Aldrige | Welsh Government | [r.aldridge@ucl.ac.uk](mailto:r.aldridge@ucl.ac.uk) |
| 38 | UK-SC | Scotland | Ian | Grant | National Statistics Department | [ian.grant@nhs.net](mailto:ian.grant@nhs.net) |
| 39 | UK-WL | Wales | Ronan | Lyons | University of Swansa | [r.a.lyons@swansea.ac.uk](mailto:r.a.lyons@swansea.ac.uk) |
