# Supplementary material for: Innovative use of data sources: a cross-sectional study of data linkage and artificial intelligence practices across European countries
Source: Arch Public Health. 2020 Jun 10;78:55. doi: 10.1186/s13690-020-00436-9 (PMC7288525; doi:10.1186/s13690-020-00436-9)
Supplement: Supplementary file 4 — Additional file 4. It is a doc. Word file and describes different data sources used for linkage across European countries in 2019. [file 13690_2020_436_MOESM4_ESM.docx]

**Additional file 4: Data sources used for linkage across European countries in 2019**

| **S/No** | **Data sources used for linkage** | | **European countries** | | |
| --- | --- | --- | --- | --- | --- |
|  |  |  | **Advanced**  **N = 24** | | **In progress**  **N = 2** |
| 1 | *Health-related administrative data sources (i.e., Electronic Health Records) ʘ* | Primary care visits, emergency care, referral records, hospital discharge, prescribed medications, health insurance claims, diagnostics procedures, laboratory tests, biobank | 22 | AT, BE, CY, CZ, DE, DK, EE, ES, FI, FR, HR, IT, LT, MT, NL, NO, PT, SI, SK, SRB, SW, UK[ENG, SC, WL] | LV |
| 2 | *Non-health related administrative data sources ǂ* | Birth and mortality database, education level, income tax, GIS, occupation, housing conditions, criminal statistics, land and housing, socioeconomic, census (demographic), house of handicap persons, environmental, road and transport, air pollution, UV light exposure | 22 | BE, CY, CZ, DE, DK, EE, ES, FI, FR, HR, IT, LT, MT, NL, NO, PL, PT, SI, SK, SRB, SW, UK[ENG, SC, WL] | IE, LV |
| 3 | *Disease-specific registries* | Cancer, diabetes, cardiovascular, congenital malformation, tuberculosis, HIV/AIDS, inflammatory bowel disease, renal, reproductive health, dementia, organ transplantation, traffic accidents/trauma or injury, hospital registry of domestic and leisure accidents | 22 | BE, BG, CY, CZ, DE, DK, EE, ES, FI, FR, HR, IE, LV, MT, NL, NO, PL, PT, SK, SRB, SW, UK [ENG, SC, WL] | LV |
| 4 | *National health surveys** | National health examination and interview surveys | 15 | BE, CZ, DK, DE, EE, ES, FI, FR, IT, NL, NO, PT, SI, SW, UK [ENG, SC, WL] | PL |
| 5 | *Population-based epidemiological cohort/National cohorts* | DANCOS, IDEFICS, CONSTANCE, ELFE, Growing up in Scotland, HealthWise Wales cohort, Millennium cohort, Caerphilly cohort study | 7 | DK, EE, FI, FR, NO, PL, UK [ENG, SC, WL] |  |
| 6 | *Clinical trials data* | FINGER, PRISMATIC | 3 | DK, FI, UK [ENG, WL] |  |

*ʘ Latvia is developing data linkage techniques to link EHRs with other data sources.*

*ǂ In Ireland, income database is linked with EHRs of prescribing medicine at small level. Latvia is developing data linkage techniques to link birth and mortality databases either with EHRs or with disease-specific registries.*

** Poland is planning to link this national health survey data with other health data sources in near future. In Ireland, this is done for specific surveys such as housing and health conditions at small scale.*
