# Supplementary material for: Innovative use of data sources: a cross-sectional study of data linkage and artificial intelligence practices across European countries
Source: Arch Public Health. 2020 Jun 10;78:55. doi: 10.1186/s13690-020-00436-9 (PMC7288525; doi:10.1186/s13690-020-00436-9)
Supplement: Supplementary file 5 — Additional file 5. It is a doc word file and describes examples of different combinations of data linkage across European countries in 2019. [file 13690_2020_436_MOESM5_ESM.docx]

**Additional file 5: Examples of different combinations of data linkages across European countries in 2019**

| **S/No** | **European countries** | **Different combinations of data linkages (N = ~ 85)** |
| --- | --- | --- |
| 1 | Austria | Hospital discharge with outpatient visit (primary care visit) |
| 2 | Belgium | Hospital discharge with health insurance claim |
|  |  | Educational attainment with mortality database |
|  |  | Census with mortality database |
|  |  | Health interview survey with mortality (cause-specific mortality, StatBEL)/use of care (IMA)/prescribed medication (INAMI) |
|  |  | Disease-specific registries with mortality database |
| 3 | Bulgaria | Registry of rare diseases with oncology registry |
| 4 | Croatia | Primary health care visits with hospital discharge/health insurance claim/mortality (cause-specific mortality) |
|  |  | Cancer registry with geospatial registry |
| 5 | Cyprus | Hospital discharge with mortality database |
|  |  | Cancer, diabetes, HIV/AIDS registries with mortality database |
| 6 | Czech Republic | Health insurance claims with mortality database |
|  |  | Registry of hospitalizations is linked with mortality database |
|  |  | Disease specific registries: Cancer, cardiovascular surgery and intervention, reproductive health, TBC registry, registry of injuries with mortality database/health insurance claims/registry of hospitalizations |
| 7 | Denmark | National patient health register is linked with education, income, housing, transfer payments, socioeconomic status, criminal statistics, etc. |
| 8 | Estonia | Hospital stay, primary and special ambulatory care linked with health insurance claims |
|  |  | Health insurance claims and prescriptions are linked with causes-specific mortality |
|  |  | Birth register linked with causes-specific mortality |
|  |  | Cancer, tuberculosis and myocardial infarction linked with causes-specific mortality |
|  |  | Chernobyl Cleanup workers (cancer, causes of death) families and children data with birth register (on irregular basis) |
|  | (In progress) | Genomic database linked with EHRs |
|  | (In progress) | Estonia health insurance database linked with prescription and diagnostics procedures |
| 9 | Finland | KANTA and KANSA health register linked with |
|  |  | Finis birth cohort 87 and 97 linked with |
|  |  | National HES from 1972-2017 linked with |
|  |  | National HIS since 1978 onwards linked with |
| 10 | France | Hospital discharge linked with health insurance claims and mortality database (national health database: SNDS) |
|  |  | Population-based epidemiology cohorts (CONSTANCES & ELFE) linked with national health database |
|  |  | Cancer, congenital malformation, cardiovascular, inflammatory bowel disease and traffic accidents registries linked with national health database |
|  |  | National health surveys (Esteban examination/interview) linked with national health database |
|  | (In progress) | UV light and air pollution exposure linked with national health database |
|  | (In progress) | House of handicap person s’ health and social assistance linked with national health database |
| 11 | Germany | National health examination survey in adults linked with mortality database |
|  |  | National health examination survey in adults linked with health insurance claims |
|  |  | Cancer registry operated by the public health institute and included in health reporting |
|  |  | National health surveys use national and sub-national data for weighting |
|  |  | National health examination surveys use inter-metropolitan socioeconomic data for improvement of field work (in progress) |
|  |  | Use of socioeconomic data at the metropolitan level for small area estimation (in progress) |
|  |  | Use of real-time emergency room data for surveillance of infectious diseases (in progress in a local project) |
|  |  | Linkage of data from national health surveys, health insurance data, cancer registry and other data sources for national burden-of-disease calculation (in progress) |
| 12 | Greece | No |
| 13 | Ireland (in progress) | Cancer registry linked with Hospital admission linked and mortality database |
|  |  | Census data linked with mortality database (one off) |
|  |  | Prescribed medication data Medical eligibility and claims data linked with income level (one off ) |
| 14 | Italy | Hospital discharge linked with mortality database and national health examination survey |
| 15 | Latvia (in progress) | Hospital discharge, primary health care, emergency care records linked with birth and mortality database |
|  |  | Patient register with specific diseases linked with mortality database |
| 1.6 | Lithuania | Compulsory health insurance information system (inpatient, outpatient specialized, primary care, emergency care) linked with causes-specific mortality database |
| 17 | Luxembourg | No |
| 18 | Malta | Health insurance claims, prescribed drugs, surgical operations, laboratory information system, radiology information system, patient administration system, outpatients attendance, patient discharge summaries linked with birth and mortality database |
|  |  | Congenital anomalies, injuries, cancer, dementia, organ transplants registries linked with mortality database |
| 19 | The Netherlands | Health examination and interview surveys linked with mortality database |
|  |  | Health insurance claims with perinatal data |
|  |  | Cancer registry data with mortality database |
| 20 | Norway | Linkage between almost all sources by means of unique personal identification. Both within health and care services, and across other governmental areas. Big data solution in use for accessibility modulation using national health registries linked with land and housing, road and transport, and GIS databases. |
| 21 | Poland | Cancer and tuberculosis registry databases linked with mortality, demographic and GIS databases |
|  | (in progress) | National health surveys linked with electronic health records |
| 22 | Portugal | Hospital discharge, primary care and medical records linked with hospital registry of domestic and leisure accidents, e-death certification |
|  |  | Cancer, tuberculosis, HIV and congenital anomalies registries linked with e-death certification and hospital discharge data |
| 23 | Romania | No |
| 24 | Slovakia | National registry of EHRs (Hospital discharge, general practitioner record, referrals, prescribed medications, laboratory results, diagnostic procedures medical consultations) linked with national disease-specific registries |
|  |  | National registry of EHRs linked with national registry of health care workers and heath care providers |
| 25 | Slovenia | Hospital discharge, drug prescription and perinatal health linked with mortality database |
|  |  | Hospital discharge, drug prescription and perinatal health linked with census data on education and socioeconomic variables (inequality analysis) |
|  |  | Hospital discharge, drug prescription and perinatal health linked with European Health Interview Survey |
| 26 | Serbia | Hospital discharge linked with cancer registry |
|  |  | Mortality database linked with cancer registry |
| 27 | Spain | National health interview survey linked with mortality database |
|  |  | Primary care data linked with drugs prescription and laboratory tests |
|  |  | National Health survey linked with cause-specific mortality data |
|  |  | All cohort studies can link their data with cause-specific mortality information through an agreement with the National Institute of statistics |
| 28 | Sweden | National Patients register linked with causes-specific mortality database |
|  |  | National Patients register linked with birth database |
|  |  | National Patients register linked with dental health database |
|  |  | National Patients register linked with vaccination database |
|  |  | National Patients register linked with education, income tax, occupation, country of origin and population based register |
|  |  | National health surveys (ULF/SILC), environmental health survey (MHE) and European health interview survey (EHIS) linked national health care quality registries (each deals with a disease-specific condition) |
| 29 | UK-England | UK Cancer Registry is linked with Hospital and Mortality Records |
|  | UK-Scotland | EHRs linked with each other: General and Psychiatric Hospital Stays/ day cases (including intensive care/high dependency stays), Outpatient attendances, Emergency department attendances, Maternity, birth records and Neonatal Care |
|  |  | EHRs linked with mortality database and census (demographic) database |
|  |  | Cancer and diabetes registries linked with hospital and mortality records |
|  |  | Scottish health interview survey linked with hospital and mortality records |
|  | UK-Wales | EHRs linked with each other: Primary care general practice datasets linked with hospital inpatient, emergency department visits, outpatient attendances, child health dataset, congenital anomalies, maternity records, population register and laboratory results in the Secure Anonymized Information Linkage (SAIL) database [www.saildatabank.com](http://www.saildatabank.com) |
|  |  | EHRs linked with mortality database, GIS and census (demographic) database |
|  |  | Cancer, trauma and renal registries linked with all of the above |
|  |  | Welsh Health Survey and National Survey for Wales (interviews) linked with all of the above |
|  |  | Healthwise Wales Cohort, Millennium Cohort, Caerphilly Cohort study and UK-Biobank linked to SAIL and all of the above |
|  |  | EHRs linked Education Attainment records and GIS derived metrics e.g. pollution, housing quality, urban design, alcohol outlets etc. |
