# Supplementary material for: Innovative use of data sources: a cross-sectional study of data linkage and artificial intelligence practices across European countries
Source: Arch Public Health. 2020 Jun 10;78:55. doi: 10.1186/s13690-020-00436-9 (PMC7288525; doi:10.1186/s13690-020-00436-9)
Supplement: Supplementary file 6 — Additional file 6. It is a doc. Word file and describes examples of health outcome indicators estimated using linked data across European countries in 2019. [file 13690_2020_436_MOESM6_ESM.docx]

**Additional file 6: Description of health outcome indicators estimated using linked data across European countries in 2019**

| **S/No** | **Categories** | **Health outcomes indicators (N = 46)** | **European countries** |
| --- | --- | --- | --- |
| **1** | **Cardiovascular (N = 14)** | | |
|  | Health characteristics | Incidence of stroke among less than and more than 65 years old | CZ, FR, LT, NL, NO, SW, UK-WL |
|  |  | Prevalence of stroke among less than and more than 65 years old | CZ, LT, NL, NO, SW, UK-WL |
|  |  | Incidence of myocardial infarction among less than and more than 65 years old | CZ, FR, LT, NL, NO, SW, UK-WL |
|  |  | Prevalence of myocardial infarction among less than and more than 65 years old | CZ, LT, NL, NO, SW, UK-WL |
|  | Mortality | Mortality due to stroke within 30-days of hospitalization | FR, IT, LV, MT, NO, SW |
|  |  | Mortality due to myocardial infarction within 30-days of hospitalization | CZ, FR, NO, SW |
|  |  | Risk of mortality due to myocardial infarction at municipal level | SW |
|  |  | Risk of mortality due to myocardial infarction at municipal level (in future) | ES |
|  | Human function and quality of life | Neurorehabilitation and functional outcomes of patients after stroke | FR, SW |
|  |  | Neurorehabilitation and functional outcomes of patients after stroke (in progress) | NO |
|  | Life expectancy and well-being | 30- days survival following stroke | FR, NO, SW, UK-WL |
|  |  | 90-days survival following stroke | SW, UK-WL |
|  |  | 365-days survival following critical care for stroke |  |
|  |  | Hospital utilization in the 365 days following intensive care discharge |  |
|  |  | 365-days survival following stroke | FR |
|  | All of above categories | DALYs, YLL, YLD | SW, UK-SC |
| **2** | **Neurodegenerative disease (N = 6)** | | |
|  | Health characteristics | Prevalence of Multiple Sclerosis | CZ, HR, FR, LT, NO, SW, UK-WL |
|  |  | Prevalence of Alzheimer | CZ, FR, LT, SW, UK-WL |
|  |  | Incidence of Alzheimer | FR, LT, SW, UK-WL |
|  |  | Incidence and prevalence of Alzheimer (in progress) | NO |
|  |  | Prior event rate ratio to estimate the influence of exposure to antipsychotic medication on acute cardiac events and hip fracture due to dementia | SW, UK-WL |
|  | Mortality | Mortality due to Parkinson | FR, SW |
|  |  | Mortality due to Dementia | FR |
| **3** | **Maternal and perinatal health/child health (N = 6)** | | |
|  | Health characteristics | Incidence of low birth weight | CY, CZ, PL, FR, LT, NO, SW |
|  |  | Incidence of pre-term birth rate | SW |
|  |  | Incidence of pre-term birth rate (in future) | CZ, FR, NO, UK-WL |
|  |  | Incidence of gestational diabetes | SW |
|  |  | Incidence of gestational diabetes (in future) | FR, NO |
|  |  | Prevalence of congenital anomalies | CZ, PT, LT, NO, SW, UK-WL |
|  |  | Emergency admissions for potentially preventable hospitalizations (PPH) between the age of 1 and 5 years | UK-WL |
|  | Mortality | Stillbirth | FR, NL, LT, NO |
| **4** | **Diabetes (N = 6)** | | |
|  | Health characteristics | Incidence of diabetes | CZ, FR, LT, SW, UK-WL |
|  |  | Prevalence of diabetes |  |
|  |  | Incidence and prevalence of diabetes (in progress) | NO |
|  | Mortality | Mortality due to diabetes and related risk factors | BG, CZ, DE, FR, SW |
|  | Human function and quality of life | Amputation rate (related complications) | BE, FR, MT, LT, SW |
|  | Human function and quality of life | Number of patients with installed insulin pump during diabetes curation | FR, PL, SW |
|  | All of above categories | DALYs, YLL, YLD | SW, UK (SC, WL) |
| **5** | **Suicide/Trauma/Injury (N = 7)** | | |
|  | Health characteristics | Use of health care services before suicide | LT, SW, UK-WL |
|  |  | Prevalence of morbid conditions before suicide |  |
|  |  | Standardized prevalence of suicide | CZ, PL, FI, LT, NO, SW |
|  |  | Incidence of suicide |  |
|  |  | Risk of road accident among users of prescribed medicines | FR |
|  |  | Incidence of injuries | LT, NO, SW, UK-WL |
|  | Mortality | Death rates in road accidents | EE, LT, NO, SW |
| **6** | **Cancer (N = 6)** | | |
|  | Health characteristics | Incidence of various types of cancer | CY, CZ, DE, FI, FR, PL, NL, NO, SW, UK-WL |
|  |  |  |  |
|  |  | Prevalence of various types of cancer |  |
|  |  | Incidence rate by stage for colorectal cancer | CZ, MT, NO, SW, UK-WL |
|  | Mortality | Mortality rates due to various types of cancer | CY, CZ, ES, FI, IT, PT, LT, NO, SW, UK-WL |
|  | Life expectancy and well-being | 5-years relative survival rates | CY, CZ, DE, EE, FI, IT, PT, NL, NO, SK, SW |
|  | Human function and quality of life | Scale of return to work after cancer and determining factors | SW |
|  |  | Scale of return to work after cancer and determining factors (in future) | BE |
| **7** | **Alcoholic liver disease and hepatic failure (N = 1)** | | |
|  | Mortality | Standardized mortality ratios at 60 days and 5-years following unscheduled admissions | SW, UK-WL |
