# Supplementary material for: Innovative use of data sources: a cross-sectional study of data linkage and artificial intelligence practices across European countries
Source: Arch Public Health. 2020 Jun 10;78:55. doi: 10.1186/s13690-020-00436-9 (PMC7288525; doi:10.1186/s13690-020-00436-9)
Supplement: Supplementary file 7 — Additional file 7. It is a doc. Word file and describes examples of health determinants identified using linked data across European countries in 2019. [file 13690_2020_436_MOESM7_ESM.docx]

**Additional file 7: Description of health determinants identified using linked data across European countries in 2019**

| **S/No** | **Domain/Health condition** | **Health determinants (N = 34)** | **Variables can be stratified by** | **European countries** |
| --- | --- | --- | --- | --- |
| **1** | **Physical environment (N = 12)** | | | |
|  | Emphysema | Air quality | Area of residence | BE, UK-WL |
|  | Injury | Place of injury | Age, sex and area of residence | CY, NO, UK-WL |
|  |  | Type of injury |  |  |
|  |  | After injury hospitalized or not |  |  |
|  | Parkinson | Exposure to pesticides (i.e., agricultural activities, in vineyards, metallurgy and solvents, in textile industry) | Area of residence | FR |
|  | Breast cancer mortality | Industrial pollution | Area of residence | ES |
|  | Adiposity | Proximity of fast food outlets from areas of residence | Area of residence | UK (ENG, WL) |
|  | Various chronic health conditions | Alcohol outlet density | Area of residence | NO, SW, UK (SC, WL) |
|  | Mental health | Presence to green-blue spaces |  |  |
|  |  | Access and visit to green-blue spaces |  |  |
|  |  | Visit to green-blue spaces |  |  |
|  |  | Housing quality |  |  |
| **2** | **Socioeconomic and environment (N = 10)** | | | |
|  | Multi-morbidity | Number of single households of older people | Age and living condition | AT, NO, SW |
|  | Breast cancer mortality, injury, diabetes, cardiovascular, mental health | Sociodemographic status | Age, sex and area of residence | EE, ES, FR, NL, NO, PT, SI, SW, UK (SC, WL) |
|  |  | Socioeconomic status |  | FR, NO |
|  |  | Employment status |  | FR |
|  |  | Level of education achieved |  | FR, NO |
|  |  | Deprivation index |  | FR |
|  | Pre-term birth | Maternal education to measure social disparities |  | FR, NO |
|  | Injury | Time and distance between road accident and emergency room |  | PL |
|  |  | Standardized absenteeism and attributable indirect costs |  |  |
|  | All types of cancer | Accessibility to linear accelerators for radiotherapy | Area of residence |  |
| **3** | **Health behavior and life style (N = 6)** | | | |
|  | Stroke, myocardial infarction, lung cancer, mental health, obesity, other chronic condition | Smoking rate | Age, sex, socioeconomic status and area of residence | BE, CY, FR, IT, MT, NL, NO, UK (SC, WL) |
|  |  | Alcohol consumption | Age and sex | NO, UK-SC |
|  |  | Physical activity |  | UK-SC |
|  |  | Dietary consumption |  | UK-SC |
|  |  | Drug use |  | NO, UK-SC |
|  | Diabetes | Diabetes risk score | Age, sex and area of residence | CY, NO, SL |
| **4** | **Biological/metabolic parameters (N = 3)** | | | |
|  | Obesity | Self-reported BMI | Age, sex and area of residence (i.e., in county, municipality) | SW, NO, UK-SC |
|  | Diabetes | Blindness | Age, sex and area of residence | CY, FR |
|  |  | Proteinuria |  |  |
| **5** | **Others (N=3)** | | | |
|  | Road accidents, neurodegenerative disease | Multi-morbidity | Age and sex | FR |
|  | Chronic health conditions | Disability |  | FR |
|  |  | Frailty |  | FR |
