# Supplementary material for: Innovative use of data sources: a cross-sectional study of data linkage and artificial intelligence practices across European countries
Source: Arch Public Health. 2020 Jun 10;78:55. doi: 10.1186/s13690-020-00436-9 (PMC7288525; doi:10.1186/s13690-020-00436-9)
Supplement: Supplementary file 8 — Additional file 8. It is a doc. Word file and describes examples of health intervention indicators estimated using linked data across European countries in 2019. [file 13690_2020_436_MOESM8_ESM.docx]

**Additional file 8: Description of health intervention indicators estimated using linked data across European countries in 2019**

| **S/No** | **Categories** | **Domain/Health condition** | **Health intervention indicators (N = 23)** | **Member States** |
| --- | --- | --- | --- | --- |
| **1** | **Maternal and perinatal health (N = 7)** | | | |
|  | Prevention | Low birth weight | Prevalence of thyroid gland examination during pregnancy | CZ |
|  | Prevention |  | Frequency of admission to intensive care unit | CY |
|  | Prevention |  | Prevalence of maternal smoking and quitting smoking during the pregnancy | FI, FR, NO, SW |
|  | Prevention | Pre-term birth | Percent of births in level III maternity units | FR, NO, SW |
|  | Prevention | Perinatal mortality | Pregnant women with adequate prenatal care (number of visit/timing of initiation) | FR, NO, SW |
|  | Prevention | Prenatal care | Screening programs as preventive check-ups during pregnancy | NO, SI, SW |
|  | Prevention | Neural tube defect | Folic acid supplementation | PT, NO, UK-WL |
| **2** | **Cancer (N = 6)** | | | |
|  | Prevention | Breast, cervical, colorectal and bowl cancer | Screening participation rates, effectiveness and evaluation | BE, EE, CZ, ES, FI, HR, IT, NO, SI, SW, UK-WL |
|  | Prevention | Colorectal cancer | Frequency of surgery | CY, NO, SW |
|  | Prevention | Colorectal cancer | Colonoscopy compliance rate | CZ, SI, NO |
|  | Prevention | Breast cancer | Genetic screening among families (in future) | ES |
|  | Prevention | Breast cancer | Stage distribution of detected cancer | CZ, SI, NO |
|  | Others | All types of cancers | Re-integration in work | BE |
| **3** | **Diabetes (N = 4)** | | | |
|  | Prevention | Diabetes related complications | Foot care | BE, FR, SW |
|  | Prevention |  | Proportion of diabetics counselled by nurse to avoid complications | SW |
|  | Prevention |  | Proportion of diabetics counselled by nurse to avoid complications (in future) | CY |
|  | Prevention |  | Amputation rate | BE, FR, SW |
|  | Prevention | Diabetes | Percentage of diabetics with latest HbA1c above 7.0 | FR, MT, SW |
| **4** | **Cardiovascular diseases (N =2)** | | | |
|  | Prevention | Stroke, myocardial infarction | Absolute global CVD risk assessment in primary prevention | IT |
|  | Prevention | Stroke | Aortic aneurysm screening | SW, UK-WL |
| **5** | **Neurodegenerative disease (N = 2)** | | | |
|  | Prevention | Multiple sclerosis | % of patients qualified for pharmacotherapy | PL |
|  | Prevention | Dementia | % of patients using neuroleptic drugs | FR |
| **6** | **Trauma/Injury/Suicide (N = 1)** | | | |
|  | Prevention | Injury/Trauma | Visit to primary care physicians before suicide | SI, LT |
| **7** | **Lower/ Upper respiratory infections (N = 1)** | | | |
|  | Prevention | COPD (Chronic Obstructive Pulmonary Disease) | % of patients with non-invasive ventilations | PL, SW |
